# Trauma Resuscitation in a Left Ventricular Assist Device Patient: An Emergency Medicine Simulation Scenario

**DOI:** 10.7759/cureus.1773

**Published:** 2017-10-13

**Authors:** William E Kenyhercz, Jorge L Pérez, Adrienne N Wolfe, Morgan R Starkey, Dominic J Bagnoli III, Ahmed F Ozgur, Anna Ciullo, Rami Ahmed

**Affiliations:** 1 College of Medicine, Northeast Ohio Medical University (NEOMED); 2 Área De Ciencias De La Salud, Instituto Tecnológico De Santo Domingo; 3 Department of Biology, Kent State University; 4 Department of Biology, The Ohio State University; 5 School of Medicine, Koç University; 6 Department of Anesthesia Critical Care, University of Cincinnati; 7 Emergency Medicine, Summa Akron City Hospital, Summa Health System

**Keywords:** lvad, emergency medicine, left ventricular assist device, simulation, simulation scenario, trauma, atls

## Abstract

Heart failure is a leading cause of death worldwide. While heart transplantation is the most successful treatment for end-stage heart failure, the scarcity in donor hearts has ushered in the use of alternative therapies, such as the left ventricular assist device (LVAD). This patient population may present with low frequency, but they require disease-specific management. Learners may fine-tune these principles in a safe learning environment, such as a medical simulation lab. Here, we present a case in which a patient with a LVAD sustained serious traumatic injuries.

## Introduction

Heart failure is a progressive clinical condition characterized by impaired filling or ejection of blood from the heart. It is further categorized by a series of stages, each of which is more severe and life-threatening than the last [1]. Recent data indicate that approximately 6.5 million people in the United States suffer from heart failure, with projections estimating a steady increase in prevalence to more than 8 million by 2030 [2-3]. In patients with end-stage heart failure undergoing traditional medical therapy, studies demonstrate one-year survival rates as low as 11%-25% [4-5]. The most successful clinical recourse in this patient population continues to be heart transplantation; however, due to a paucity in the availability of donor hearts, alternative treatments, such as left ventricular assist devices (LVADs), are becoming increasingly more common [6].

left ventricular assist device (LVAD) is a device that assists or completely replaces the function of a failing heart by functionally connecting the left ventricle directly to the aorta to manually circulate blood via a battery-powered pump [7-8]. LVADs are primarily used as a bridge to transplantation for eligible patients, but they can also be used as a permanent therapy in patients ineligible for transplantation [7]. Current reports demonstrate one-year survival rates as high as 80% in all LVAD patients [9]. While LVADs were historically used as a bridge mechanism, promising one-year survival rates have led to an increase in long-term use [10]. Due to the growing success and versatility of LVADs in helping treat end-stage heart failure, patients receiving LVADs have increased from just 98 in 2006 to over 2,000 per year between 2012-2014 [9].

Despite the increasing prevalence of patients with LVADs, there is a dearth of research on how these patients should be properly managed in the emergency department [11-12]. Given the delicate and intricate nature of the LVAD device and its function, patients may not present with expected vitals on clinical assessment (LVAD patients typically do not have peripheral pulses and their blood pressure cannot be measured using automated cuffs), and some traditional advanced cardiac life support methods (e.g., chest compressions) must be avoided in order to preserve the function of the LVAD and circumvent any additional iatrogenic injury [7]. Considering the increasing burden of heart failure as well as the concomitant rise in LVAD placement, it is of utmost importance that healthcare providers in emergency settings be well trained in managing patients with LVADs. Therefore, the purpose of this simulation is to guide clinicians in readily recognizing and managing a trauma patient with a LVAD device in order to improve patient outcomes.

## Technical report

**Figure 1:**
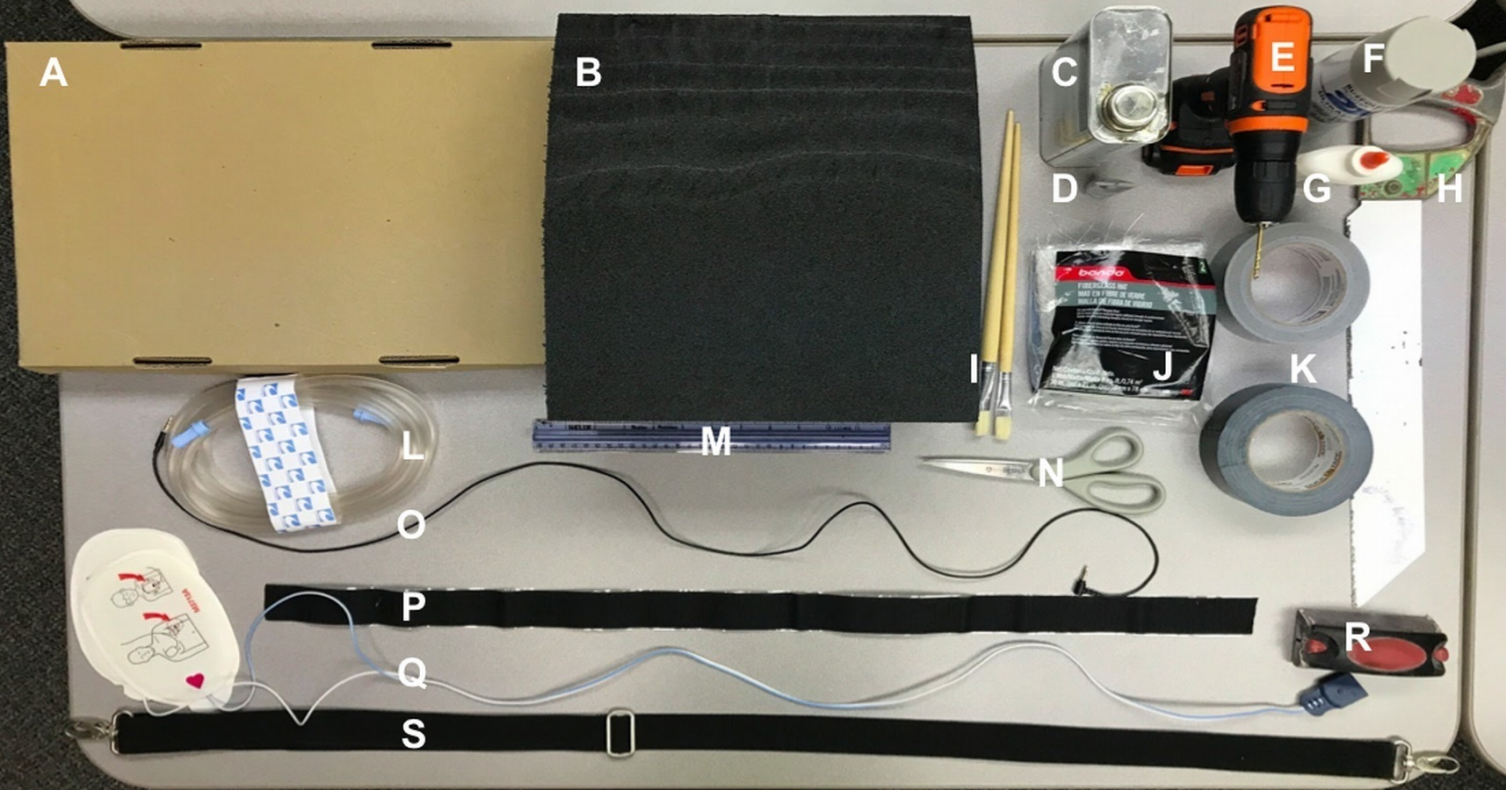
Simulated left ventricular assist device (LVAD) materials list A. Cardboard; B. Foam packing material; C. Fiberglass resin; D. Liquid hardener; E. Drill; F. Paint; G. Glue; H. Saw; I. Paintbrushes; J. Fiberglass; K. Duct tape; L. Standard suction tubing; M. Ruler; N. Scissors; O. Auxiliary cord; P. Velcro; Q. Wiring from defibrillator pads; R. Sandpaper; S. Briefcase strap

The LVAD trauma case is simulated to take place in a community emergency department. The team has access to standard equipment, medications, and consultants. The simulation is performed with a Simulaids Stat Manikin with Deluxe Airway Management Head (Simulaids, Inc., Saugerties, NY) wearing a simulated LVAD modeled after the HeartWare LVAD (HeartWare, Framingham, MA). The simulated LVAD consists of two batteries, a control unit, a pump, inflow tube to the pump from the left ventricle, outflow tube to the aorta from the pump, and a driveline cable (Figure 1). The manikin is fitted with a belt that holds the LVAD control unit and both batteries. The LVAD driveline cord is attached to the manikin via tape to the chest area. This simulation provides an opportunity to manage a trauma patient with a LVAD device and to assess the learner’s ability to appropriately manage a high-risk, low-frequency scenario.

Replication of the LVAD

Necessary materials:

1. Simulaids Stat Manikin with Deluxe Airway Management Head simulator

2. Briefcase strap

3. Foam packing material

4. Sandpaper

5. Cardboard

6. Ruler

7. Saw

8. Duct tape

9. Scissors

10. Standard suction tubing

11. Wiring from a defibrillator

12. Auxiliary cord (x2)

13. Velcro

14. Glue

 

Optional materials:

1. Fiberglass

2. Fiberglass resin

3. Liquid hardener

4. Paint

5. Paintbrush (x2)

6. Drill

 

**Figure 2:**
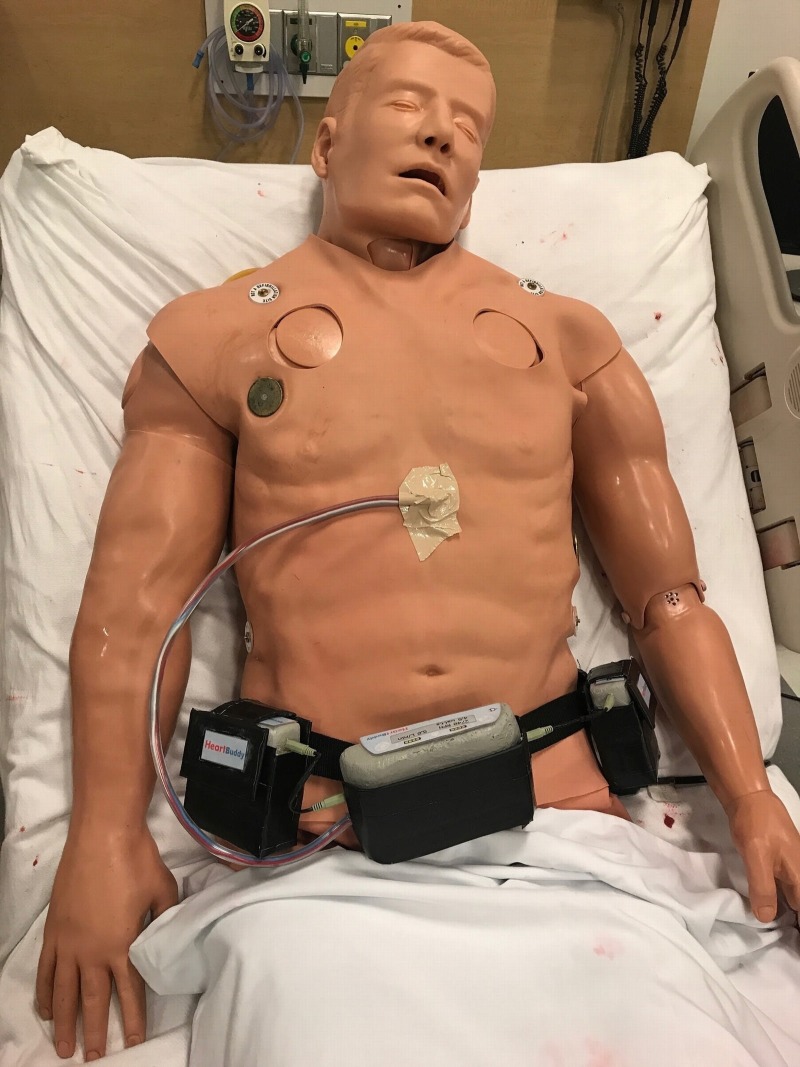
Simulaids Stat Manikin with Deluxe Airway Management Head Simulator wearing simulated left ventricular assist device (LVAD)

**Figure 3:**
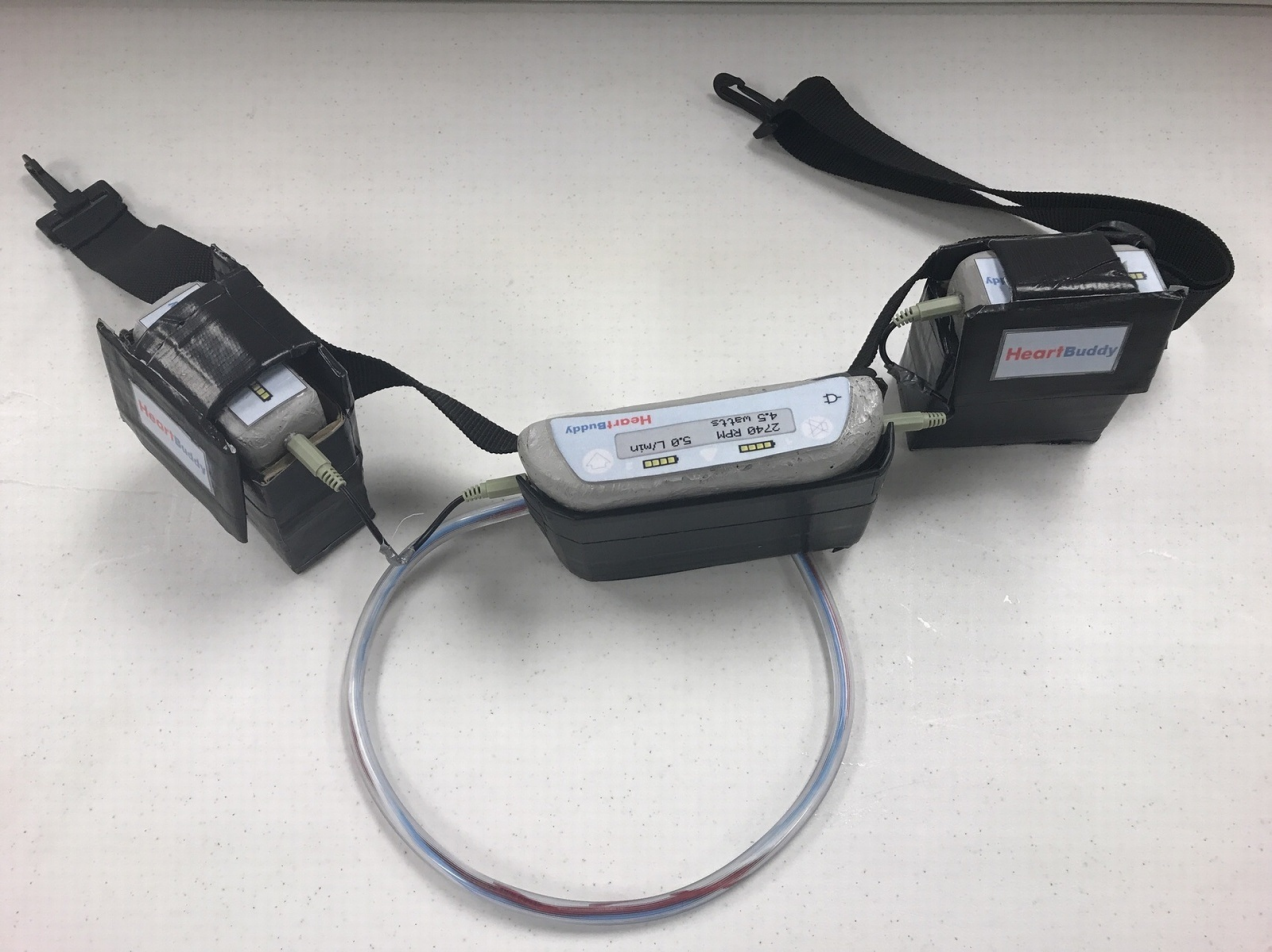
Final simulated left ventricular assist device (LVAD) as per the materials listed in Figure 1 and replication instructions

Application:

1. Cut the foam into two “LVAD battery packs” (9.5 cm x 4 cm x 8 cm) and one “LVAD controller” (9 cm x 4 cm x 13 cm) (5 min)

2. Sand the pieces of foam to smooth the edges and form appropriate shapes (5 min)

3. OPTIONAL: Use fiberglass resin and liquid hardener to imitate the plastic feel of the device (application: 15 min, set time: 2 hours, sanding: 30 min)

4. OPTIONAL: Paint the batteries/controller for a more realistic appearance (application: 15 min, set time: 1 hour)

5. Cut cardboard to form two battery cases and a case for the controller (25 min)

6. Apply one side of the velcro to the back of the controller and battery cases and the other side to the briefcase strap (all 13 cm apart) to form the “LVAD belt” (5 min)

7. Hollow out one small hole in each battery; hollow out two small holes and one medium hole in the controller. If fiberglass is applied, a drill will be needed to make holes (5 min)

8. Put one end of the auxiliary cord in the small hole located on the battery and the other end in the controller. Repeat step for second battery; use glue to hold if needed (5 min)

9. Insert defibrillator wiring into standard suction tubing (5 min)

10. Attach one end of standard suction tubing into the medium-sized hole in controller; use glue to hold if needed (5 min)

11. Wrap the LVAD belt around the waist of the simulator and attach the other end of the standard suction tubing to the chest of the simulator (Figures 2-3).

After the gathering of materials is complete, creating the simulated LVAD should take approximately one hour. The optional steps increase completion time to five hours; however, the finished product will more accurately portray the actual device. The device will also be more durable and usable for a longer period of time.

Preparing the simulation scenario

**Figure 4:**
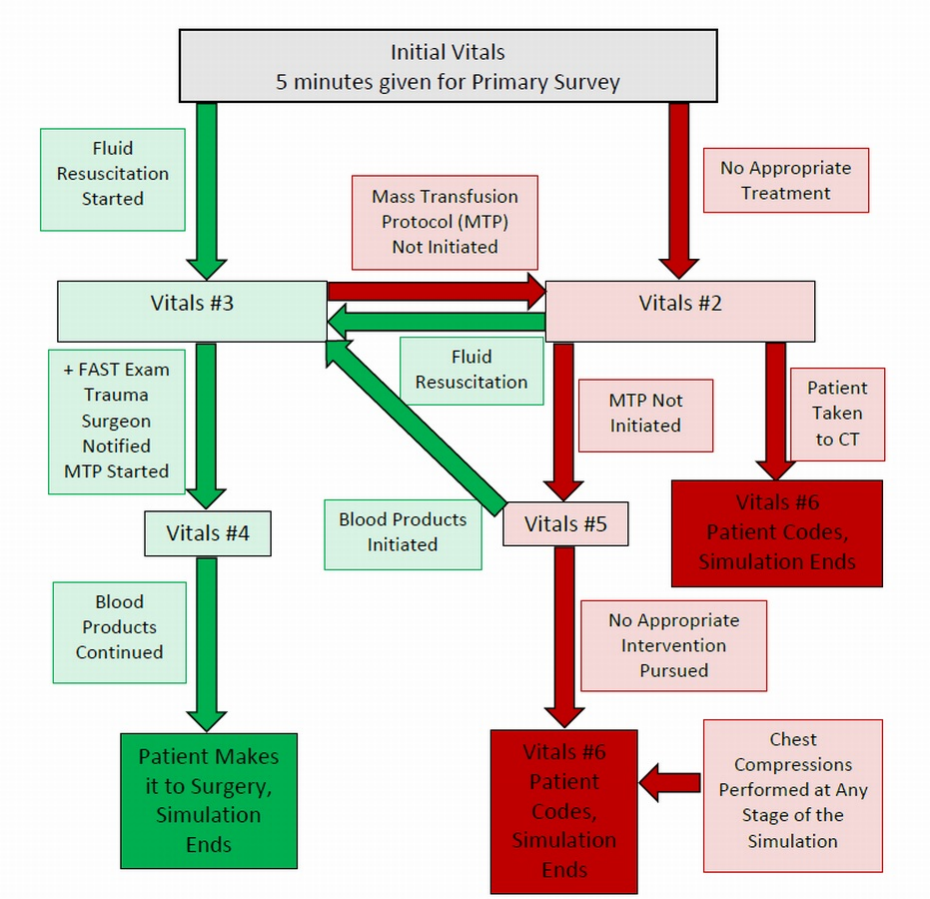
Vitals flow chart (branch points) Vitals numbers in the figure correlate with the vitals listed in Table 1.

The staff involved in the simulation includes a confederate nurse, a confederate emergency medical technician (EMT), a simulation technician, and at least one faculty member. The faculty are pre-briefed and provided with an outline of the complete case, including patient history, critical decision points, and vitals and labs (Tables 1-2). Throughout the case, the simulator’s vitals are adjusted to reflect the management decisions of the learners as outlined in the flowchart (Figure 4), with the green lines illustrating the proper pathway and the red lines illustrating the improper pathway.

**Table 1:** Branch point vital signs MAP = mean arterial pressure RR = respiratory rate RA = room air *See Figure 4 for corresponding anticipated branch points in patient management

	Heart Rate	MAP (if obtained)	Temperature	Oxygen Sats (RA)	RR
#1	112		37	94%	26
#2	122	55	37	94%	26
#3	106	65	37	98%	24
#4	100	75	37	98%	22
#5	144	45	37	91%	26
#6	20	0	37	69%	Agonal

**Table 2:** Laboratory values for simulation scenario INR = international normalized ratio EtOH = ethanol WBC = white blood cells Hb = hemoglobin Hct = hematocrit PLT = platelets CO2 = bicarbonate level BUN = blood urea nitrogen * Note INR of three consistent with expected anticoagulation of left ventricular assist device (LVAD) patient

Complete Blood Count	Basic Metabolic Panel	Lactic Acid	Liver Function Test	INR	Tox Screen	Urinalysis	EtOH
WBC: 12,000/microL (4,000-11,000)	Sodium: 138 mEq/L (136-145)	1.5 mg/dL (0-1.5)	Within normal limits	3 (2-3)	Negative	Normal	Negative
Hb: 10 mmol/dL (12-15)	Potassium: 4.2 mEq/L (3.5-5.1)						
Hct: 27 mmol/dL (36-45)	Chloride: 98 mmol/L (98-107)						
PLT: 150/microL (100-250)	CO2: 24 mmol/L (22-30)						
	BUN: 18 mg/dL (6-20)						
	Creatinine: 0.9 mg/dL (0.6-1.4)						
	Glucose: 135 mg/dL (70-100)						

**Figure 5:**
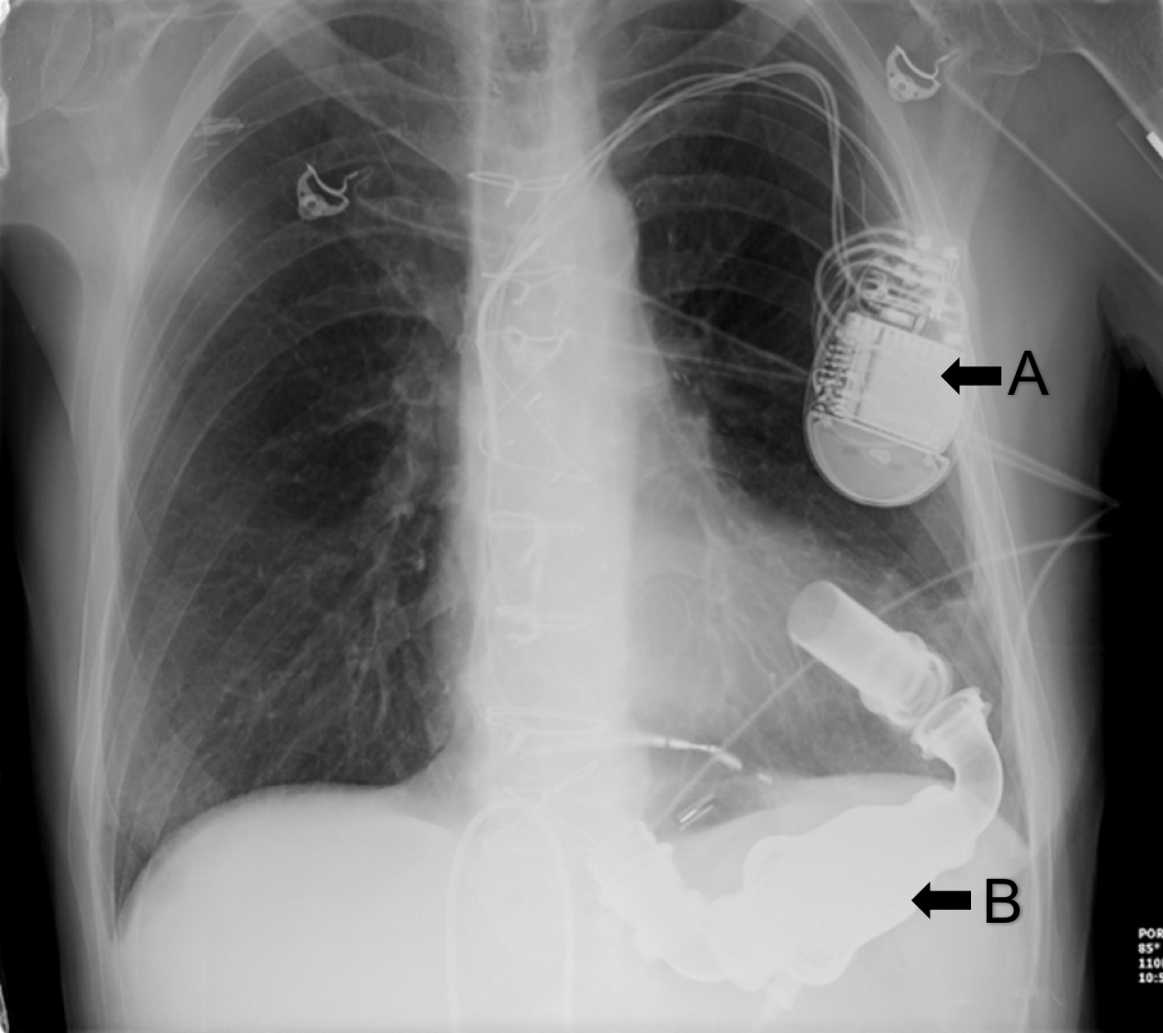
Chest x-ray showing left ventricular assist device (LVAD) A = implantable cardioverter defibrillator (ICD) B = left ventricular assist device (LVAD) Courtesy of: https://emrems.com/tag/lvad/

**Figure 6:**
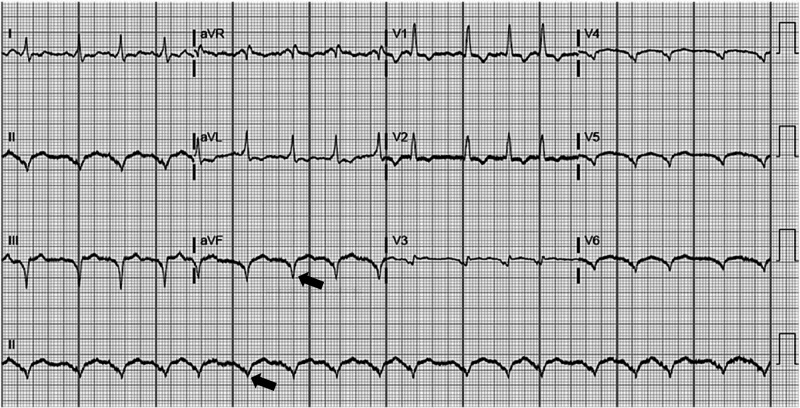
EKG demonstrating sinus tachycardia in a patient with a left ventricular assist device (LVAD) * Note QRS duration > 120 ms consistent with left ventricular assist device (LVAD) placement as indicated by the arrows Courtesy of: http://www.sciencedirect.com/science/article/pii/S0735675717301675#f0015

Images of a chest x-ray (CXR), electrocardiogram (EKG), and focused assessment with sonography in trauma (FAST) exam (Figures 5-6) are chosen by the faculty and included in the simulation. A technician provides the patient’s voice through the manikin to facilitate physician-patient communication. The details of the case are standardized and include patient prescription medications, patient medical history, and the lab work ordered by the learners. A confederate nurse is present to enact orders and communicate information to the learners upon request.

Pre-briefing

A pre-briefing meeting was held prior to the start of the case, in which the learners were instructed to address the simulation as if it were a real-life situation. The extent of the manikin’s capabilities was discussed along with the resources available for treatment and the roles of the staff in the simulation. The learners then conferred to delineate each of their roles ahead of the simulation.

Case

A 63-year-old male presents via Emergency Medical Service (EMS) after being involved in a two-car motor vehicle collision. EMS reports that the patient was a restrained passenger when he was struck by an oncoming car traveling at approximately 50 mph. On the scene, the patient was alert and oriented but complained of abdominal pain. Medics were unable to obtain a blood pressure reading but remaining vitals are stable. Upon arrival at the emergency department (ED), the patient continues to complain of left upper quadrant (LUQ) abdominal pain while denying any chest and neck/back pain, shortness of breath, headache, or neurological deficits. The patient has a medical history of stage IV chronic heart failure, hyperlipidemia, and diabetes mellitus. Patient's surgical history includes a pacemaker, cholecystectomy, and a LVAD placed four months ago. Patient takes warfarin, insulin glargine, insulin lispro, and atorvastatin. Patient reports no known drug allergies.

The case is considered successfully managed if the learners obtain a focused history from EMS (and/or the patient), appropriately execute a primary survey, initiate standard therapy, and recognize the LVAD as the reason for the lack of blood pressure and pulse. Learners should remain focused on the resuscitation of the patient and not be distracted by the LVAD, identify a positive FAST exam and initiate the massive transfusion protocol (MTP), order an EKG and CXR, attempt to call the LVAD coordinator within five minutes, and notify the trauma surgeon. If the patient is taken to a CT scanner at any time (with persistently unstable vital signs), MTP is not initiated, and/or chest compressions are performed, the patient codes and the case is terminated after a few additional minutes per the discretion of the senior faculty.

Debriefing

A debriefing session was held for all simulation participants upon completion of the case. The technique for the discussion incorporated a theory-based framework based on a combination of critical feedback and inquiry [13]. Faculty facilitated learners’ identification and analysis of their own performance, their communication with and management of an interprofessional staff, and their development of a broad differential diagnosis.

The main discussion points focused on the management of a LVAD patient who has suffered an acute traumatic injury with a normally functioning LVAD in comparison to the management of a typical trauma patient, an overview of which can be found in Table 3. The ability to recognize a LVAD and treat the underlying pathology via a standard primary survey while taking care to avoid interfering with the operation of the device itself was paramount in the debriefing.

**Table 3:** Key differences in typical patients vs. left ventricular assist device (LVAD) patients LVAD = left ventricular assist device MAP = mean arterial pressure VF = ventricular fibrillation VT = ventricular tachycardia RPM = revolutions per minute

TYPICAL PATIENT	LVAD PATIENT
Perform primary survey	Call LVAD coordinator and perform primary survey
Chest compressions if needed	No chest compressions; compressions performed as a last resort if all else has failed
Pulse should be present	Pulse weak or absent
Find arterial blood pressure	Find MAP (goal 70-90 mmHG); if MAP difficult to auscultate, insert arterial line for continuous measurement
Pulse oximetry present	Pulse oximetry typically present
Can auscultate patient to hear heartbeat	Auscultate LVAD to hear a “hum” (left upper quadrant of abdomen)
Use defibrillator if needed	If VF or VT present, apply defibrillator but not over the LVAD
No driveline	Check for infections around driveline
May or may not be anticoagulated	Anticoagulated
VF and VT with no pulse results in absent vitals and unresponsiveness	VF and VT without a pulse can present with a measurable MAP and fully-conscious patient
	Check to see if LVAD has power and the controller is properly connected, do not cut the LVAD wire
	Check heat of skin around LVAD, abnormality may indicate clot in pump
	Check RPM and flow on LVAD

The standard primary survey served as the initial focus of the discussion, as many learners frequently failed to follow advanced trauma life support (ATLS) protocol or were very distracted by the LVAD at the outset of treatment, delaying critical resuscitative management. The discussion then focused on the LVAD device itself and the delicate procedure for treating a LVAD trauma patient. Learners were initially confused about the exact function of the LVAD and had limited or no knowledge of the importance of calling the primary LVAD coordinator to better understand the mechanics and physiology of the LVAD and the relevant precautions that need to be followed. Perhaps as a result of a lack of understanding, or a misunderstanding of the device’s function, learners were not familiar with typical vital signs and physical exam findings that often normally occur with a LVAD patient. Specifically, the difficulty in obtaining pulse and blood pressure readings presented a significant obstacle to the learners, who were not aware that mean arterial pressure (MAP) had to be taken to measure blood pressure when traditional measurements were unreliable, and that the pulse may be very diminished or non-palpable [7]. Additionally, learners initially had trouble understanding that a LVAD patient in ventricular fibrillation (VF) and ventricular tachycardia (VT) could still be responsive secondary to the continuous hemodynamic support provided by the LVAD.

Post-scenario didactics

Upon completion of the debriefing, all learners participated in a 20-minute didactic session to reinforce the key teaching points during the simulation. Importance was placed on differences in methods of proper management of a typical patient when compared with a LVAD patient. In addition, the importance of first checking the LVAD and contacting the LVAD coordinator was emphasized. The discussion of the management of a LVAD patient focused on obtaining the MAP, following ATLS protocol, and the importance of fluid for hypotensive trauma victims regardless of other extraneous circumstances. Following the lecture from senior simulation faculty, time was given for the learners to ask questions.

## Discussion

Heart failure is a common medical condition that is affecting the US and the broader global population at an alarming rate. While heart transplantation remains the most clinically successful means to treat end-stage heart failure, LVAD devices have become an increasingly common and effective form of alternative or permanent treatment, suggesting a forthcoming rise in the frequency with which these devices are seen. This increase in this patient population will, therefore, necessiate the training and education of emergency medicine providers [14]. In our experience, many emergency medicine physicians are ill-equipped to manage the nuances of treating a LVAD patient effectively in a trauma scenario. Due to the severe consequences of interfering with a functioning LVAD, simulating an emergency scenario is an invaluable method to teach learners about the management of such patients without clinical repercussions for the patient. Our simulation is structured to emulate a trauma case with a LVAD device, wherein learners may face some of the potential pitfalls and complications intrinsic to working with and around these devices.

Additionally, the case was meant to serve as an ATLS primary survey review. Successful resolution of the scenario was therefore contingent upon prompt recognition of the LVAD and appropriate resuscitation of the patient’s traumatic injuries while remaining mindful of navigating the LVAD device. Our previous experiences demonstrate that learners consistently struggle with certain aspects of this particular case as revealed in the debriefing. Notably, the failure of learners to execute a proper ATLS primary survey secondary to the distraction (and sometimes bewilderment) of the LVAD device is problematic. Many learners immediately identify the LVAD device, coupled with the notification from the confederate nurse that the patient has no palpable pulse and no blood pressure, yet the patient is speaking and complaining of abdominal pain. Many learners spend several minutes discussing the LVAD with the patient or approaching the patient as if they were walking into a room with a stable patient complaining of abdominal pain. After several minutes of mismanagement (lack of aggressive fluid resuscitation and proceeding down the primary survey), the patient becomes unresponsive, which often results in the patient being resuscitated as a typical cardiac arrest patient, with chest compressions, leading to iatrogenic dislodging of the device.

With respect to the implications of the device itself in a trauma scenario, learners failed to recognize the importance of involving the LVAD coordinator, which is a vital management step to resuscitating this delicate patient. Likely owing to their unfamiliarity with the device itself, learners were unaware of the benefits of involving the coordinator, such as guidance in troubleshooting, operation, and what steps to take to properly treat the patient. Learners also had difficulty obtaining valuable vital sign information to help them guide their resuscitative efforts. Namely, learners struggled to acquire a blood pressure reading, which is a regular occurrence in LVAD patients and is critical to guide the fluid resuscitation of a trauma patient. Furthermore, the finding of diminished or absent pulse confused the learners, who expected a patient presenting with no pulse to be unresponsive. Additionally, a number of clinically deleterious iatrogenic complications may arise, resulting in LVAD disruption, such as rapid exsanguination if the pump is dislodged. In order for clinicians to adequately treat LVAD patients presenting with trauma, they should have a fundamental understanding of the basic function, physiology, and troubleshooting of the LVAD device to attain optimal clinical outcomes in this patient population.

## Conclusions

This case underscores the importance of recognizing and safely working around a LVAD device in a trauma simulation scenario. Learners are mentored to adhere to ATLS protocol while remaining cognizant of the device and the potential complications that may arise as a result of improper management.
